# Advancing Care in Alzheimer’s Disease: Current Treatments and Their Impact on Quality of Life

**DOI:** 10.7759/cureus.90527

**Published:** 2025-08-19

**Authors:** Leyla Yashaeva, Melissa Montoya, Juee Joshi, Aliyah Stephens, Olivia McNulty, Gurjinder Kaur

**Affiliations:** 1 Basic Biomedical Sciences, Touro College of Osteopathic Medicine, Middletown, USA; 2 Physiology, Touro College of Osteopathic Medicine, Middletown, USA

**Keywords:** alzheimer's disease, amyloid plaques, cognitive decline, dementia treatment, disease-modifying drugs, fda-approved therapies, neurodegeneration, tau protein

## Abstract

Alzheimer's disease (AD) is a progressive neurodegenerative disease characterized by memory deficits and cognitive decline. It is currently the most common cause of dementia globally and carries a significant disease burden, given the rising aging population worldwide. Although AD is debilitating for the patient, the complex nature of the disease is also a source of tremendous mental distress for caregivers. Finding, understanding, and evaluating the best treatments can be challenging and overwhelming when coping with a life-changing diagnosis of AD. Although there is currently no definite cure for AD, all the discussed treatments aim to improve a patient's quality of life and attenuate the time spent in disability. This study compiles and discusses the risks, benefits, and efficacy of all currently available treatments for AD, including treatments that target the control of cognitive and non-cognitive symptoms. Additionally, the manuscript discusses investigational therapeutics, including small molecules and immunotherapies in phase 2 or 3 clinical trials, and assesses non-pharmacological interventions that may alleviate disease burden.

## Introduction and background

Alzheimer’s Disease (AD) is a crippling neurocognitive disorder that affects many worldwide. It is currently responsible for 60-80% of all cases relating to dementia. Approximately 50 million individuals worldwide are currently affected by AD, which is expected to double every five years, reaching an estimated 152 million people with AD by 2050 [1]. Cognitive impairments, such as the gradual loss of memory and the decline of thinking abilities, distinguish AD. Additionally, behavioral and psychological impairments, such as hallucinations, delusions, anxiety, sleep disturbances, and restlessness, are also frequently noted. Fundamentally, AD is characterized by extracellular deposition of amyloid-beta (Aβ) plaques with abnormal buildup of intracellular neurofibrillary tangles (NFTs) or tau proteins as a result of protein misfolding. Accumulation of these proteins contributes to neuronal toxicity. It causes premature cell death through increased oxidative stress and disruption of the cell cycle and synaptic transmission, potentially playing a crucial role in the neuropathology of AD [2-4].

The prominence of the Aβ peptide has been a source of great interest in the study of AD, making it a target for therapeutic interventions [5]. The Aβ peptide is derived from the β-amyloid precursor protein (APP), which is processed by α- and γ-secretases. Processing by these secretases leads to the production of soluble polypeptides that are further broken down and processed by the cell [5-7]. In AD, however, APP is processed by β- and γ-secretases, resulting in the production of insoluble Aβ peptides. These insoluble peptides aggregate and form plaques that disrupt neuronal signaling, resulting in AD-associated memory loss [5-7]. Moreover, plaque formation is known to be associated with cerebral amyloid angiopathy and inflammation that damages neurons [5-7]. Cerebral amyloid angiopathy is characterized by the buildup of Aβ plaques on the lumen of cerebral arteries, leading to hemorrhage or rupture [8,9]. Aβ is transferred across the blood-brain barrier from brain-to-blood via a receptor-mediated transcytosis process involving the LRP1 receptor [10]. Implication of either of these proteins can also lead to the accumulation of Aβ in the brain, a known histological hallmark of AD. Some attention has also been directed toward the catabolic process that Aβ undergoes [11]. Aβ degradation is carried out by neprilysin and insulin-degrading enzymes. Studies have noted a diminishment of both proteins with normal aging and AD-vulnerable regions of the brain [11]. These studies suggest the potential role that an imbalance between the production and degradation of Aβ plays in the progression of AD.

AD is also characterized by the accumulation of intracellular NFTs that accumulate inside the cytoplasmic structures of neurons [12]. If hyperphosphorylated, the tau protein, which normally plays an important role in the stabilization of the microtubules and their packing structure, leaves the microtubule aggregates, leading to the formation of NFT [9,13]. In the 1980s-1990s, paired helical filaments (PHF) were found in NFTs, and tau protein, a major microtubule-associated protein, was identified as the main PHF within these mature neurons [14-16]. It is suggested that the Aβ plaques trigger the activation of the kinases, causing the phosphorylation of the tau protein. Tau’s interaction with the microtubules is essential for its signaling function, and an impairment in signaling may lead to neuron apoptosis, followed by brain shrinkage and atrophy [9,13]. The atrophy, on the other hand, causes characteristic narrowed gyri and broadened sulci associated with AD. Similar to Aβ plaques, NFTs are considered synaptotoxic, and their presence is associated with cognitive decline in AD patients [9,13].

Carriers of the apolipoprotein E4 (APOE4) gene are considered to be at higher risk for the development of AD. APOE4 carriers have an earlier and more extensive Aβ plaque deposition in the brain and increased tau phosphorylation compared to APOE2 and APOE3 carriers. APOE2 carriers, on the other hand, have a delayed onset of Aβ plaque deposition, less severe pathology, and more intact cognitive ability. It shows that the apoE protein plays an important role in the neuropathology of AD, and gaining a better understanding of the APOE protein’s impact gives potential for the development of new therapeutic therapies modulating APOE levels [17-19].

A significant effort was made in the development of the AD treatments currently available on the market. Despite the inability to fully cure AD yet, progress in alleviating the symptoms of AD and lessening its burden has already been achieved. Three main categories can be identified amongst the current drugs and therapies for AD. The first category of drugs is used for the treatment of cognitive symptoms of AD. There are currently three approved anticholinesterase inhibitors: donepezil (Aricept®), galantamine (Razadyne®), and rivastigmine (Exelon®). Such drugs are used for the treatment of individuals with all types of dementia, mild-to-moderate dementia, and moderate-to-severe dementia, respectively. Memantine (Namenda®), an N‐methyl‐D‐aspartate (NMDA) receptor antagonist, can also be used for the treatment of moderate-to-severe dementia.

In some cases, memantine is used in combination with donepezil (Namzaric®). The second category of drugs targets non-cognitive symptoms. The orexin receptor antagonist, suvorexant (Belsomra®), was approved for the treatment of insomnia in patients with AD. Alternatively, brexpiprazole (Rexulti®), an atypical antipsychotic drug affecting dopamine and serotonin chemical pathways, was approved for the treatment of agitation in AD patients. The third category of drugs, anti-monoclonal antibodies, can alter the progression of AD. Drugs such as aducanumab (Aduhelm®) and lecanemab (Leqembi®), for example, are indicated for mild cognitive impairments and dementia [20-22].

This review aims to summarize current FDA-approved therapies, investigational agents, and non-pharmacological approaches, with a focus on their impact on quality of life.

## Review

Treatments targeting cognitive symptoms

The types of cognitive symptoms that may arise in AD are dependent upon an individual’s disease stage. These include, but are not limited to, issues with the recollection of names and events, recognition of familiar people, and formulation of plans [23,24]. The following drugs cannot prevent neuronal cell damage, as is evident throughout the course of AD; however, the use of such treatments can potentially reduce or stabilize the progression of cognitive symptoms associated with memory and thinking capabilities [22]. Examining various targets, including cholinergic dysfunction, glutamatergic excitotoxicity, amyloid and tau aggregation, neuroinflammation, and synaptic degradation, will facilitate a deeper understanding and connection with AD.

Cholinergic Dysfunction

Acetylcholine is an excitatory neurotransmitter that plays a vital role in learning and memory [25]. Brain regions, such as the hippocampus, are densely populated by nicotinic acetylcholine receptors. The loss of such receptors underlies the pathophysiology seen in AD [25]. The cholinergic hypothesis postulates that a loss of such receptors, and a consequential decrease of intracellular acetylcholine levels, leads to cognitive decline [26]. Drugs such as galantamine, donepezil, and rivastigmine are current therapy options for AD that increase acetylcholine levels in the synaptic cleft by inhibiting the enzyme acetylcholinesterase (AChE). AChE carries out the degradation of acetylcholine within the synaptic cleft, enabling cells to return to their resting state following an action potential [27]. Inhibitors of AChE prevent hydrolysis and degradation of acetylcholine, thus increasing its availability within the synaptic cleft [28]. Although AChE inhibitors do not prevent the progression of AD, they have been proven to be effective at alleviating symptoms of mild to moderate AD [25].

Galantamine: Galantamine is the most commonly used AChE inhibitor amongst those in existence. Galantamine, a plant alkaloid, functions as both a competitive and reversible AChE inhibitor [29]. Although galantamine was extracted and characterized during the early 1950s, it was not approved by the FDA until 2001 [30]. Today, it is marketed as Razadyne® and used for treating varying diseases, such as vascular dementia, Parkinson’s disease, Lewy body disease, adult autism, schizophrenia, dementia related to multiple sclerosis, bipolar disorder, and post-traumatic nerve palsy. Galantamine’s anti-inflammatory properties have also been studied in diseases such as diabetes and metabolic syndrome disorder [31].

Galantamine currently exists in an oral form and is characterized by immediate or extended release. Gastrointestinal adverse events are commonly cited with the usage of this medication, specifically abdominal pain, diarrhea, vomiting, and nausea [32]. Such side effects, however, are typically mild and associated with the usage of higher dosages. Galantamine may also cause cardiovascular effects such as atrioventricular blocks and sinus bradycardia [32].

Donepezil: Donepezil shares the same mechanism of action as galantamine. As a reversible inhibitor of AChE, donepezil accentuates cholinergic transmission [33]. Its usage in mild, moderate, and severe dementia has been found to alleviate symptoms of AD by improving cognition [33]. Donepezil’s therapeutic role has extended to include off-label uses in conditions such as Lewy body dementia, traumatic brain injury, dementia in the context of Parkinson’s disease, and vascular dementia [33]. Recent findings have also highlighted its potential role in the downregulation of neuroinflammatory responses that occur in AD [33]. A meta-analysis conducted in 2022 analyzed a total of 17 studies to gauge the effectiveness of donepezil. Upon the use of donepezil, participants were noted to display improvement in both the mini-mental state examination (MMSE) and the Montreal Cognitive Assessment scales [34]. Despite such improvements, donepezil did not have the capability of halting the progression of the disease itself [33,34].

Donepezil is currently available in multiple forms, including transdermal and oral forms. Similar to galantamine, donepezil may be associated with gastrointestinal side effects. These include, but are not limited to, nausea, vomiting, and diarrhea. Other commonly cited side effects include muscle fatigue, nausea, and dizziness [35]. A more dire consequence of donepezil is the occurrence of cardiac arrhythmias, such as Torsades De Pointes. However, in such cases, this can be reversed upon the cessation of donepezil [36].

Rivastigmine: Rivastigmine, marketed as Exelon®, is a selective AChE inhibitor that treats dementia in patients with AD and Parkinson’s disease [37,38]. Anticholinesterases are drugs that block the breakdown of acetylcholine into acetic acid and choline, in turn increasing the amount of acetylcholine in the nervous system [25,26]. Acetylcholine is a neurotransmitter that is important for learning and memory. There are two cholinesterase enzymes in the human body, and they are capable of hydrolyzing acetylcholine: AChE and butyrylcholinesterase [25,26]. The former is mostly found at the synapses and in the areas of high activity within the cerebral cortex. In contrast, the latter is in the glial cells and helps with the management of cholinergic activity [25,26]. With age, the activity of cholinesterase enzymes increases; however, it increases even more in AD and Parkinson’s disease patients [25,26]. Dementia progresses when the synaptic connection between the basal forebrain and cortex or hippocampus is lost, leading to memory loss [25,26]. Rivastigmine possesses the ability to reversibly bind to and inhibit AChE and butyrylcholinesterase and inhibit their activity, leading to increased levels of acetylcholine and enhanced cholinergic transmission in the brain [25,37-40].

Rivastigmine is a drug indicated to treat mild-to-moderate dementia caused by AD and was approved by the FDA in 1997 [37]. It is currently available in the forms of oral capsules, oral solution, and transdermal patches. Rivastigmine has good blood-brain barrier penetration; moreover, compared to other anticholinesterases, cytochrome P-450 is not involved in its metabolism, leading to fewer drug-drug interactions [37,41,42].

Alpha-secretase is a metalloprotease that cleaves APP in the Aβ domain, thus preventing the production of toxic Aβ and producing neuroprotective and neurotrophic sAPPα fragments [43]. Research shows that rivastigmine is capable of sAPPα elevation and reduction in concentrations of potentially neurotoxic sAPPβ and Aβ [43]. The neurotoxicity arises from sAPPβ’s ability to activate microglia [44].

Thus, rivastigmine can affect the alpha-secretase pathway and early stages of AD. Since its efficacy and tolerability have already been investigated, with some optimization, rivastigmine can be an impactful treatment for dementia patients, not only via its main anticholinergic effects [43,45].

Side effects mostly include gastrointestinal problems, including nausea and vomiting, as well as rare cases of stomach ulcers, hyporexia, episodes of acute cholinergic syndrome, and dysphagia [37,46-48]. Gastrointestinal side effects are more rarely seen with the transdermal route of administration. Less frequently, adverse effects may include weakness, sleep cycle disturbances, and muscle cramps. Patients using transdermal patches are more prone to having contact dermatitis [37].

Meta-analysis investigating the efficacy of oral and transdermal patch rivastigmine in 19 clinical trials with 7445 patients has demonstrated that the drug is significantly better than a placebo in delaying functional impairment [41,49-51]. Patients in the higher dose group showed an increase of four points or more compared to placebo [50]. Functional impairment is a diagnostic criterion for AD and dementia. It is the functional decline in complex activities such as bathing, dressing, eating, and transferring, which are known as activities of daily living (ADL) [52].

The study analyzing seven trials, including 3450 patients with mild-to-moderate AD, proved that rivastigmine led to better cognitive outcomes when compared with placebo [41,53]. Also, research using positron emission tomography (PET) as well as magnetic resonance imaging has shown an increase in metabolism in the hippocampus in patients taking the drug. The transdermal patch’s efficacy was studied in 1195 patients and was compared to capsules of rivastigmine and placebo. The research led to a conclusion that the efficacy of the 10 cm² patch is similar to the efficacy of capsules, whereas its side effects are close to those of a placebo [41,54]. A meta-analysis that used data from 6611 AD patients in 36 clinical studies investigated the impact of donepezil, rivastigmine, galantamine, and memantine. It suggests that besides benefits in stabilizing and slowing the decline of cognitive function and functional outcome in patients with mild-to-moderate AD, rivastigmine is effective in patients with moderate-to-severe and severe AD [55].

Glutaminergic Excitotoxicity

Excessive release of glutamate, known as glutaminergic excitotoxicity, leads to neuronal damage and activation of excitatory plasma membrane receptors [56]. Mechanisms associated with excitotoxic cell death include the pro-death signaling cascade of glutamate receptors, calcium overload, mitochondrial impairment, excessive glutamate within the synaptic cleft, and altered energy metabolism [56]. The NMDA receptor (NMDAR) is a glutamate receptor; glutamate, in turn, is a primary excitatory neurotransmitter in the human nervous system. The NMDAR and excitatory activity of glutamate play an important role in synaptic plasticity, but the excessive receptor activity, glutamate signaling, and Ca2+ entry lead to excitotoxicity and a potential for cell death, which explains the degeneration of the neurons in AD patients and subsequent loss of their cognitive abilities [57-59].

Memantine: Memantine, marketed as Namenda®, is an NMDAR antagonist indicated for the treatment of the moderate to severe cognitive symptoms of AD [22,60]. Memantine is a non-competitive NMDAR antagonist that possesses low to moderate affinity to the receptor and binds to the channel on the NMDAR, inhibiting further binding of the glutamate [61]. The action of memantine resembles the action of the positive ion of magnesium on the NMDAR, which also has an inhibitory effect on excitotoxicity caused by the overactivation of the receptor [62]. Memantine has no efficacy in treating mild symptoms in patients with AD; however, since 2003, the drug has been approved by the FDA for the treatment of moderate-to-severe AD [60,63,64].

Common side effects caused by the usage of memantine are headache, confusion, dizziness, drowsiness, hypertension, hallucinations, restlessness, diarrhea, and constipation [60,61]. Non-adherence to medications is common among patients with neuropsychiatric diseases, and in the developed world, adherence to long-lasting treatment therapies is only about 50% [65]. Since AD patients with cognitive impairments might have a reduced awareness of being non-compliant with the memantine treatment regimen, the oral form, which has gastric retention equivalent to seven daily memantine treatments, has been developed. The drug has been tested in animals, and in dogs, the newly developed form has nearly 100% bioavailability and has a matrix formulation delivering medication without any food requirements throughout the week [66].

Memantine has been used as monotherapy for 20 years. Throughout this time, as studies were taking place, the positive effects of memantine have been shown. Clinical studies prove that memantine indeed leads to improved cognitive, functional, and behavioral test results in AD patients compared to placebo [67,68]. In six 24-/28-week, randomized, double-blinded studies, participants had moderate to severe AD [69]. Patients received memantine 20 mg/day or received a placebo. At weeks 12 and 24/28, the behavioral endpoint was evaluated using the neuropsychiatric inventory (NPI), a clinical instrument for assessing a range of behavioral and psychological symptoms in people with dementia or other neurological conditions [69]. At week 12, among patients symptomatic for hallucinations at baseline, 68% of those treated with memantine showed improvement in the NPI hallucinations item compared to 49% receiving placebo [69]. In patients who were asymptomatic for specific NPI domains at baseline, a significantly higher proportion remained symptom-free compared with placebo [69]. At week 12, this benefit was observed for agitation/aggression (86% vs 79%), delusions (90% vs 87%), and disinhibition (92% vs 88%) [69]. At week 24/28, memantine continued to show a protective effect, with more patients remaining asymptomatic for agitation/aggression, irritability/lability, and nighttime behavior disturbances [69].

These results highlight memantine’s role not only in improving existing behavioral symptoms but also in preventing the emergence of new neuropsychiatric issues over both short- and longer-term follow-up [69]. Cognitive symptom improvement caused by memantine is also demonstrated in the study, where a 24-hour sleep deprivation mimicking working memory impairment in AD patients and consequent worsened reaction time and memory performance was resolved with the usage of memantine [70]. In addition, studies show that hippocampus avoidance during whole-brain radiation therapy plus memantine can preserve cognitive abilities in patients with brain metastases [68,69]. Hippocampus avoidance means that the doses are limited to the bilateral hippocampal dentate gyri during whole-brain radiation therapy, which could prevent any associated cognitive toxicity [68]. On the other hand, multiple meta-analysis studies, including the one involving 5004 patients and 18 clinical studies, have shown that despite its beneficial effect on cognitive abilities and good toleration among AD patients, the drug has a small efficacy outcome and, as a result, a small clinical benefit if used as monotherapy [68,71,72].

AD is associated with a decrease in the production of hydrogen sulfide (H₂S) by the central nervous system [73]. H₂S is a neuromodulator interacting with NMDAR that seems to regulate the levels of calcium ions intracellularly [73]. Moreover, it possesses neuroprotective, anti-apoptotic, and anti-inflammatory properties, including the reduced release of IL-1 and IL-6 induced by amyloid β-peptides, as well as upregulation of COX2 and activation of NF-kβ. Thus, these qualities of H₂S may become beneficial in treating cognitive impairment and neurodegeneration in AD patients [74-76]. Based on these findings, a memantine prodrug was proposed; a free amine group was replaced with an H₂S-generating moiety called memit. The prodrug was studied in human neuron-like cells and rat microglia cells, which led to the conclusion that the drug indeed protects neurons from oxidative stress and cytotoxicity, decreases neural injury and pro-inflammatory cytokine release, causes a reduction in Aβ accumulation, and reduces the damage produced by Aβ oligomers. Although more in vivo studies are needed to further investigate the memantine prodrug, it represents the first drug capable of producing H₂S in the central nervous system from a previously developed drug, memantine [76].

Combination Therapy Cholinergic Dysfunction and Glutamatergic Excitotoxicity: Memantine + Donepezil

In contrast to monotherapies, combined therapies are advantageous given their ability to target multiple molecules simultaneously. Today, memantine and donepezil are commonly used as monotherapies or in combination for the treatment of AD. As an AChE inhibitor, donepezil works synergistically with memantine, which functions as an NMDA receptor antagonist [77]. Memantine/donepezil combined therapy exists under the brand name of Namzaric®. Namzaric® was approved by the FDA in 2014 for patients suffering from moderate-to-severe AD [78,79].

At higher dosages, Namzaric® usage may be associated with weight loss, vomiting, and syncope [80]. According to the study involving the Japanese Adverse Drug Event Report database, from April 2004 to May 2022, there were 6718 arrhythmia cases out of 333,702 reported cases for anti-dementia drugs [81]. Since anticholinesterases increase levels of acetylcholine, their adverse effects on the heart are expected [81]. The association between heart-related side effects and memantine is not well known, though. Nevertheless, there are case reports demonstrating adverse cardiovascular effects caused by memantine. A significant association with arrhythmia was shown with the combination of drugs as well. As a result, after prescribing memantine and donepezil, a continuous follow-up of AD patients with adverse effect monitoring may be especially needed [81].

A meta-analysis studied the usage of memantine, donepezil, and combination therapy in individuals with moderate-to-severe AD, and findings revealed that combination therapy was the most effective at improving ADL, enhancing cognition, and alleviating neuropsychiatric symptoms [78,82]. Each showed a significant difference compared to placebo based on the Alzheimer’s Disease Assessment Scale-Cognitive Subscale (ADAS-cog) and Severe Impairment Battery (SIB) scales [69]. The authors concluded that the combination therapy was better than the monotherapy, and the placebo had the worst effect on cognition among all options according to ADAS-cog and SIB [69]. Behavioral disturbances were rated using the NPI, and patients who were symptomatic experienced improvement with memantine compared to placebo in 8 out of 12 individuals [69]. A separate meta-analysis of nine studies comprising 2,604 patients specifically compared combination therapy with anticholinesterase monotherapy in patients with MMSE scores below 20 [82]. In the short term, combination therapy produced a significantly greater improvement in cognitive function; however, no significant difference was observed in ADL or behavioral and psychological symptoms [81]. Importantly, the addition of memantine did not increase the incidence of adverse effects, indicating a comparable safety profile between combination therapy and monotherapy [82].

Treatments targeting non-cognitive symptoms 

Understanding the relationship between AD, tau pathology, and circadian rhythm dysfunction is crucial for developing targeted interventions to treat non-cognitive symptoms, such as managing sleep disturbances and behavioral symptoms in affected individuals. Treatment of these non-cognitive symptoms is important because they can lead to early institutionalization and accelerated caretaker burnout [83,84]. Current therapeutic approaches include strategies to help improve behavioral symptoms, such as agitation, and better regulation of the circadian rhythms in AD patients [85,86].

Agitation and regulation of circadian rhythms in AD are both multifactorial non-cognitive symptoms, involving a complex interplay of neurobiological and chemical interactions. One notable neurotransmitter for drug regulation of non-cognitive symptoms is serotonin. Its critical role within the central nervous system is highlighted by its abundant presence within the central nervous system [87,88]. Serotonin plays a crucial role in synaptic plasticity and acts as a neuromodulator, influencing the direct effects of other neurotransmitters [87,88]. Specifically, serotonin inhibits dopamine release and modulates the transmission of glutamate and GABA [87,89]. In the hippocampus, serotonin receptors decrease glutamate release and promote the release of GABA from inhibitory interneurons, thereby reducing long-term potentiation [87,89]. Conversely, in the frontal cortex, serotonin inhibits glutamate release, while in the prefrontal cortex, it enhances glutamate transmission [87,89,90]. Consequently, serotonin deficiency or dysregulation in Alzheimer's dementia may directly impact cognitive and non-cognitive symptoms, as well as disrupt its modulation of other neurological systems.

Notably, in the rat brain, each cortical cell is closely associated with a neighboring serotonin-containing neuron, underscoring the widespread influence of serotonin modulation [88,91]. Additionally, a study using a radiotracer [11C]‐3‐amino‐4‐(2‐dimethylaminomethyl‐phenylsulfanyl)‐enzonitrile ([11C]‐DASB) demonstrated that individuals exhibiting non-cognitive behavioral or psychiatric symptoms had a reduced number of serotonin transporters in both cortical and limbic brain regions when compared to those without such symptoms [88,92]. Another study found that decreases in serotonin transmission or postsynaptic receptor densities are associated with several symptoms seen in Alzheimer's dementia, including aggression, impulsivity, increased appetite, and depression [88,93,94]. Moreover, dysregulation of these systems may contribute to agitation in AD. The prevalence of non-cognitive behavioral and psychiatric symptoms within Alzheimer's dementia patients was found to be 97%, and these symptoms have been associated with worse prognosis and earlier death [83,84,92,95,96]. Thus, highlighting the importance of understanding these underlying mechanisms to develop effective strategies for managing agitation in individuals with AD.

AD presents with many aspects of cognitive decline and various degrees of neuropsychiatric symptoms, such as agitation. Agitation is known to be one of the most complex and difficult aspects of care for patients with Alzheimer’s dementia [97]. One FDA-approved drug, brexpiprazole (Rexulti®), can be used for the treatment of agitation in Alzheimer’s dementia. Brexpiprazole functions as a partial agonist at 5-HT1A and dopaminergic D2 receptors [86,98]. Additionally, it acts as an antagonist at noradrenergic α1B and α2C receptors, as well as serotonergic 5-HT2A receptors [86,98]. In two double-blind studies, brexpiprazole 2 mg/day showed significant clinical improvement in treating agitation in Alzheimer's dementia patients when compared to placebo trial groups [97,99]. A third trial that required patients to meet the International Psychogeriatric Association definition of agitation at entry, which was not available at the time of the previous trials, also found a significant difference in treating agitation with brexpiprazole 2 mg and 3 mg when compared to placebo groups [98].

In addition to abnormal behavioral symptoms, the human sleep-wake cycle is also dysregulated in AD. Prolonged periods of sleep deprivation or wakefulness accelerate the accumulation of AB plaques and tau proteins in the brain [100-106]. Conversely, during sleep, our bodies effectively clear toxic metabolites like Aβ and tau [107,108]. This accumulation of Aβ plaques and tau proteins directly correlates with disturbances in the sleep-wake cycle dysregulation in AD patients [85,104,107-109]. The combination of these findings, coupled with the suggested role of sleep in memory consolidation and learning, highlights the importance of regulating sleep as an additional therapeutic strategy in neurodegenerative disorders such as AD. Hospital-acquired delirium is a common and serious complication among patients with AD. In a three-year, community-based cohort study of individuals with AD, up to two-thirds experienced at least one hospitalization, and delirium occurred in approximately one-quarter of all patients during these hospital stays [110]. Patients who developed delirium in the hospital had higher risks of death, nursing home placement, and faster cognitive decline compared to those hospitalized without delirium [110]. In this study, dementia severity was assessed using the Massachusetts General Hospital Dementia Severity Rating scale, and AD was the primary diagnosis in the majority of participants [110]. These findings underscore both the high prevalence and the profound impact of hospital-acquired delirium in these patients [110].

One neuropeptide that can be densely found within the brain is orexin, which has projections in the serotonergic dorsal raphe nucleus, noradrenergic locus coeruleus, and histaminergic tuberomammillary nucleus, and all of these nuclei are involved in promoting arousal [111]. Orexin specifically promotes wakefulness through the OX1R and OX2R receptors [112]. It is also known that antagonizing these orexin receptors increases sleep [113-115]. Additionally, it was shown that mice lacking orexin had decreased Aβ pathology in the brain and an increase in sleep time compared with mice expressing orexin [104]. Thus, making orexin receptors a valuable target to inhibit to help improve the sleep cycle in AD patients. Therapeutic agents such as suvorexant (Belsomra®) target these orexin receptors to help combat insomnia in AD patients. Suvorexant is a known dual orexin receptor antagonist and was recently found to successfully decrease both tau phosphorylation and Aβ plaque concentrations in the brain [85]. In three randomized, double-blind, placebo-controlled clinical trials, suvorexant showed significant increases in total sleep time by 73 minutes compared to patients in the placebo trial groups, exemplifying its usefulness in treating insomnia in AD patients [112,116,117].

Treatments targeting disease progression

Amyloid and Tau Aggregation: Anti-amyloid Monoclonal Antibodies

Anti-Aβ monoclonal antibodies are disease-modifying treatments based on the “amyloid hypothesis,” which proposes that aggregation of Aβ plaques triggers a downstream cascade of pathological events leading to neuroinflammation, synaptic dysfunction, and formation of phosphorylated tau tangles [118]. Phosphorylated tau tangles contribute to Aβ toxicity via a feedback loop that enhances synaptic dysfunction and neurodegeneration, therefore causing the cognitive decline seen in AD patients [119]. The binding of monoclonal antibodies to Aβ aggregates facilitates their clearance from the brain, thus reducing their direct and toxic downstream effects and slowing further cognitive decline. There are multiple monoclonal antibodies in development; however, only donanemab and lecanemab are currently approved by the FDA. Although they aim to achieve the same results, each monoclonal antibody targets different species of amyloid beta plaques [120].

Lecanemab, marketed as Leqembi®, is a recombinant Aβ monoclonal antibody distinct from donanemab, as it is highly selective against soluble amyloid protofibrils and moderately selective against insoluble fibrillar amyloid plaques, making it an ideal combination targeting the most pathogenic of the amyloid species in AD [120]. It was promoted from accelerated to traditional approval by the FDA on July 6th, 2023, after a confirmatory trial with approximately 1800 participants, which verified the treatment’s benefits [121]. Over an 18-month period, participants receiving the drug showed a significant reduction in amyloid burden and slower progression of cognitive and functional decline compared with placebo, as measured by the ADAS-Cog and Alzheimer’s Disease Cooperative Study-Activities of Daily Living scales [121]. The primary efficacy outcome was a 27% relative reduction in the rate of decline on the Clinical Dementia Rating-Sum of Boxes (CDR-SB) over 18 months (95% CI: 14-39%; p < 0.01) compared to placebo [121]. Thus, on average, disease progression, as captured by the CDR-SB, was slowed by over one-quarter over a year and a half [121]. Although lecanemab has a clear, significant effect on cognition and total amyloid burden, the clinical effects are hardly perceivable to the patient or caregiver. They are significantly less than other available cholinesterase inhibitors such as donepezil [122]. However, lecanemab is anticipated to give patients greater benefit over time since it modifies disease progression as opposed to only controlling symptoms. The half-life of brain amyloid re-accumulation as measured by amyloid PET was estimated to be approximately four years, suggesting that it will take approximately 16-20 years (4-5 half-lives) for brain amyloid to reaccumulate and return to its value before treatment [123]. Since lecanemab prolongs the early stage of dementia, clinical trial data show substantial evidence in reducing AD patients’ disability-adjusted life years, allowing them to function independently and alleviating the emotional and physical stress faced by many caregivers when caring for late-stage dementia patients.

Once approved for treatment, lecanemab is administered every two weeks for approximately one hour and monitored for any infusion reactions or amyloid-related imaging abnormalities (ARIA) post-infusion with an MRI after the 4th, 7th, and 14th infusions. An additional MRI at week 52 is recommended for patients who are APOE4 carriers or those with evidence of ARIA on previous MRIs [124].

Donanemab, marketed as Kisunla®, is another humanized monoclonal antibody derived from mice mE8-IgG2a that uniquely targets deposited Aβ plaques in the brains of patients with AD. It is the second monoclonal antibody to receive traditional FDA approval in July 2024 after reassuring clinical results of the phase 3 trial TRAILBLAZER-ALZ2 [125]. The trial consisted of about 1700 participants with symptomatic mild cognitive impairment/dementia with low to medium or high tau protein burden as evident on PET imaging and cognitive assessments. Participants were observed over 76 weeks to assess any change in integrated Alzheimer's Disease Rating Scale (iADRS) score and secondary measures such as CDR-Sum of Boxes, MMSE, ADAS-Cog13, repeat PET and MRI, and AD biomarkers. Cognitive and functional decline slowed in all measures, especially by 35% on the iADRS in patients with low to medium tau and by 22% in the combined analysis of all participants [126]. Separate data for the high-tau group in this trial were not provided. However, in a study comparing the effects of aducanumab and donanemab, participants above a 1.46 standardized uptake value ratio of tau protein threshold (high tau) showed no benefit in reduction in AD biomarkers after plaque clearance, indicating that donanemab may not be beneficial for patients with moderate to severe dementia [127]. In addition, in patients with low/medium tau, 47% showed no disease progression for at least one year compared to 29% on placebo. The study showed that for patients with mild dementia from AD, donanemab slowed disease progression by 32% over 18 months, and the difference was significant [126]. In this trial, “disease progression” was defined as the rate of change on the integrated iADRS from baseline to week 76 [126]. This one-year no-progression outcome was a separate categorical result and was not used to calculate the modeled delay values [126]. The 4.36-month (iADRS) and 7.53-month (CDR-SB) values represent the estimated additional time it would take patients receiving donanemab to reach the same level of decline as the placebo group, based on modeling of the rate of decline observed over the 76-week trial period [126].

For comparison, lecanemab reduced cognitive decline by 27% (95% CI: 14-39%, p < 0.01) compared to placebo over 18 months [126]. The primary outcome measure evaluated the change in baseline on iADRS from baseline to 76 weeks [126]. Moreover, in contrast to the lecanemab trial, participants' treatment was switched to placebo when brain scans showed complete clearance of tau proteins from the brain [128]. While it reflects donanemab’s specificity to deposited tau protein, its implementation is essential in decreasing patients' and their families’ hospital burden, cost, and unnecessary treatment [128]. Phosphorylated tau 217 blood tests and brain amyloid PET scans were used to monitor disease progression throughout the trial and have been suggested for monitoring beyond the 76 weeks of the trial [128]. Guidelines for clinicians for monitoring AD patients post-stopping treatment are currently being studied.

Donanemab and lecanemab have the same indications adopted from profiles of subjects in the drugs’ respective clinical trials. Patients of any age can be considered for this treatment if they meet all other criteria. To be eligible, patients must undergo a full neuropsychological panel to inspect for MCI or the mild dementia stage of AD with confirmed Aβ pathology on amyloid/tau PET scan or other cerebrospinal fluid AD biomarkers, such as elevated P-tau or A𝛽42 [129]. MMSE scores of 22-30 were required for patients to participate in the lecanemab clinical trials [129]. It is also important that one can tolerate closed-sided MRIs, as they are needed initially and throughout treatment to examine contraindications. Patients must also be counseled on APOE4 genotyping and the extent of ARIA risk prior to starting treatment. The incidence of ARIA was dose-dependent and highest in homozygous carriers of the APOE4 gene, with donanemab having a lower incidence compared to lecanemab [130].

Contraindications to monoclonal anti-Aβ antibody treatment include lacunar infarcts, past hemorrhage on MRI, more than four microhemorrhages, superficial siderosis, uncontrolled seizure disorder, anticoagulation medications, poorly controlled psychiatric illnesses, or any other neurological disorders contributing to the patient’s MCI [124]. Lecanemab has been shown to increase the risk for brain hemorrhage with acute thrombolytic treatment [124]. A particular incidence of cerebral hemorrhage and necrotizing vasculopathy leading to death was reported following a tissue plasminogen activator infusion for an acute ischemic stroke in a patient receiving lecanemab [131]. Although antiplatelet medications, such as aspirin or clopidogrel, are not a contraindication for treatment, their use may increase the risk of ARIA in patients homozygous for the APOE4 gene [124]. Pregnant or breastfeeding females are excluded, and females of child-bearing age must be using contraception as both drugs have not been tested for their teratogenic or reproductive side effects [125]. Lastly, subjects with a body mass index less than 17 and greater than 35 or a score higher than 8 on the Geriatric Depression Scale are recommended to be excluded, as done in phase 2 and 3 trials for Lecanemab [124]. Lastly, monoclonal anti-Aβ antibody treatment is not indicated for other diseases that cause cognitive impairment, such as Lewy body dementia, Parkinson’s disease, frontotemporal dementia, and vascular dementia [124,125,131].

Treatment can be stopped in case of the development of contraindications, placement of an MRI-noncompatible pacemaker, patient preferences, caregiver decisions, or the physician’s recommendations based on careful risk-benefit analysis [121]. Both lecanemab and donanemab must be stopped in all patients showing stroke-like symptoms and seizures with the presence of ARIA [121]. In other ARIA-related complications, treatment can be suspended and resumed depending on whether ARIA-E/H stabilizes or resolves. The FDA provides more detailed information about dosage interruptions to guide clinicians in their judgment [121]. Moreover, since neither antibody has been tested in patients with moderate or severe dementia, any progression into the advanced stages of AD calls for reassessment of treatment continuance and physician-patient agreement on the risk/benefit ratio of resuming treatment. Moderate dementia is indicated by CDR global scores of 2.0, MMSE scores below 20, and inability to perform ADLs [129].

**Table 1:** Current FDA-approved treatments for AD AchE: acetylcholinesterase, NMDA: N‐methyl‐D‐aspartate, Aβ: amyloid-beta, AD: Alzheimer's disease, ARIA: amyloid-related imaging abnormalities, FDA: Food and Drug Administration

Drug name	Target(s)	Therapy-associated benefit(s)	Potential adverse effects	FDA approval
Donepezil	AchE inhibitor	Treatment of dementia in mild, moderate, and severe AD	Nausea, vomiting, diarrhea, muscle fatigue, dizziness	1996
Rivastigmine	AchE inhibitor	Mild to moderate dementia in patients with AD	Nausea, vomiting, stomach ulcers, hyporexia, episodes of acute cholinergic syndrome, dysphagia	1997
Galantamine	AchE inhibitor	Mild to moderate severe AD	Mild: abdominal pain, diarrhea, vomiting, nausea. Moderate to severe: atrioventricular blocks and sinus bradycardia	2001
Memantine	NMDA receptor antagonist	Moderate to severe cognitive symptoms of AD	Headaches, confusion, dizziness, drowsiness, hypertension, hallucinations, restlessness, diarrhea, constipation	2003
Memantine + donepezil	NMDA receptor antagonist and AchE inhibitor	Moderate to severe AD	Cardiovascular side effects	2014
Lecanemab	Soluble amyloid protofibrils and insoluble fibrillar amyloid plaques	Reduces markers of amyloid in early AD and reduces the decline in cognitive functioning	Infusion-related reactions: headache, chills, and fever. Anger, cognitive disorders, disorientation, abnormal dreams	2023
Donanemab	Aβ plaques	Reduces markers of amyloid in early AD and reduces the decline in cognitive functioning	ARIA	2024

Non-pharmacological interventions

The inclusion of non-pharmacological interventions is an important aspect of this review, as these approaches can enhance the quality of life for individuals with AD. The manuscript highlights three promising interventions: cognitive stimulation therapy (CST), structured exercise programs, and multi-domain lifestyle modifications, which may complement pharmacological treatments to support brain health.

Cognitive Stimulation Therapy

CST is a structured, evidence-based psychosocial intervention employed in the management of dementia, including AD, to enhance patients’ quality of life [132]. CST typically involves engaging individuals within a small group form to promote cognitive engagement and social interaction through stimulating activities, including memory tasks, problem-solving exercises, and group discussions [133]. Evidence demonstrates that CST yields significant improvements in short-term global cognitive function and behavioral symptoms in individuals with mild to moderate dementia [132]. Furthermore, a systematic review encompassing 26 studies concluded that CST is associated with improved cognitive performance and a reduction in depressive symptoms in this patient population [134].

Structured Exercise Programs

Structured exercise programs are increasingly recognized as a valuable non-pharmacological intervention for individuals with dementia, particularly for mitigating neuropsychiatric symptoms such as depression, agitation, and apathy [135]. In a hospital-based randomized controlled trial, the intervention group demonstrated a significant reduction in neuropsychiatric symptoms, with the most notable improvement observed in agitation [135]. Agitation, as measured by both emotional and psychomotor scales, showed a significant change with the NPI and Cohen-Mansfield Agitation Inventory surveys [132]. A change of 11 points in the NPI and a change of 8 points in the Cohen-Mansfield Agitation Inventory is clinically relevant, and the intervention group showed -11 for NPI and -10 for CMAI [135]. Incorporating brief physical activity may be especially beneficial for patients residing in nursing homes or those who are bedridden, as immobility can exacerbate stress and agitation [136].

Multi-domain Lifestyle Interventions

Multi-domain lifestyle interventions are being studied heavily for patients with dementia and AD. Multi-domain lifestyle interventions are modifiable risk factors that intervene in at least two different domains: one corresponding to nutritional/diet and one additional intervention including cognitive training, physical exercise, social activity, or vascular risk management [137]. The Finnish Geriatric Intervention study investigates a two-year multidomain lifestyle intervention on cognitive functioning [138]. Baseline measurements showed sufficient potential for prevention [138]. Multi-domain lifestyle interventions are a holistic approach for AD to reduce the risk or slow the progression of AD. Additionally, it allows for a patient-centered approach through modifiable factors as discussed in the Finnish Geriatric Intervention study.

More research is needed on non-pharmacologic interventions; however, initial studies for all three subsets have shown promising results in supporting cognitive function and improving neuropsychiatric symptoms that may arise in patients. This more personalized and holistic approach, specialized for each patient, can be a valuable tool to mitigate cognitive decline.

Investigational therapies in phase 2 and 3 clinical development

The current therapeutic pipeline for AD is ongoing with many targets, especially with the advances in immunotherapy. While it is difficult to include all the ongoing trials, two potential treatments in phase 3 trials have been depicted to illustrate the variety of targets and deliveries of treatments. Phase 3 trials were prioritized over small studies because they are larger and have progressed further than phase 1 or 2. NE3107 has been included to discuss a small molecule targeting neuroinflammation, and ACI-35 is an immunotherapeutic vaccine that targets tau.

NE3107 (Bezisterim) is a small molecule targeting neuroinflammation in a phase 3 clinical trial for mild-moderate AD [139]. NE3107 is a derivative of beta-androstenteriole, which has been modified to increase oral bioavailability and stability by binding to extracellular signal-regulated kinase ½ (ERK1/2) kinases and is blood-brain barrier permeable [139]. ERK1/2 is involved in inflammatory signaling and insulin responses; however, it has no interaction with the steroid hormone receptors. Additionally, ERK inhibits inflammation driven by ERK and NF-κB pathways, including TNF-α [139]. The study objectives utilize the Alzheimer’s Disease Assessment Scale Cognitive Subscale 12 and the Alzheimer's Disease Cooperative Study-Clinical Global Impression Change over 30 weeks [139]. Results from November 2023, where NE3107 was compared to placebo, may be equal to or greater than the benefit from approved AD monoclonal antibodies, and patients had a 4.66-year age deceleration [139]. The most common adverse event associated with NE3107 is a mild headache that was reported in 10% of treated patients [139]. The analysis utilized 57 patients out of 439 enrolled due to protocol violations; however, the company is beginning another phase 3 trial in 2025 to be given orally once a day.

ACI-35 (JNJ-64042056) is a liposome-based vaccine targeting tau that is in a phase 2/3 clinical trial [140]. ACI-35 induces an immune response of pathological conformers of phosphorylated tau, but it does not elicit an autoimmune response against physiological forms [140]. The vaccine has 16 copies of synthetic tau fragments, which are phosphorylated at the pathological residues S396 and S404 within a lipid bilayer and the mono-phosphoryl-lipid A adjuvant [140]. The study enrolled 500 cognitively healthy individuals who had preclinical AD with amyloid and tau biomarker positivity [141]. Cognitive decline is being assessed and is measured by the Preclinical Alzheimer’s Disease Cognitive Composite 5. Results from the phase 1b/2a clinical trial in 2022 showed that all participants mounted an antibody response to phosphorylated tau, and many participants had antibodies to PHFs and non-phosphorylated tau [141]. Additionally, no significant adverse events have been reported, and the treatment has been well tolerated [141].

Emerging strategies targeting tau and neuroinflammation, as discussed above, will be critical for AD patients and help to alleviate the disease progression. It will be critical to see the clinical data as both studies progress and further correlate it with our discussion around quality of life.

## Conclusions

AD is a progressive neurodegenerative disorder characterized by cognitive and memory impairments. Multiple pathological mechanisms contribute to the progression of AD, including cholinergic dysfunction, glutamatergic excitotoxicity, Aβ and tau protein aggregation, neuroinflammation, and synaptic degradation. Genetic factors also play a significant role, with carriers of the APOE4 allele exhibiting a higher risk for developing the disease. Although no cure currently exists, substantial efforts are underway to expand the therapeutic pipeline, aiming to target these underlying mechanisms, alleviate symptoms, and improve patients’ quality of life.

Current interventions for AD include both pharmacological and non-pharmacological approaches, as discussed in this review. Most of the available pharmacological treatments focus on managing cognitive decline. Recent research, such as the Finnish Geriatric Intervention study, has explored the potential benefits of non-pharmacological interventions, highlighting the importance of a holistic treatment approach. Ongoing and future clinical trials will be critical to evaluating the efficacy of emerging therapies, including small molecules and immunotherapies, and their potential to reduce the overall burden of AD. Additionally, it is imperative for future studies to research head-to-head drug comparisons, biomarker validation, and targeted therapies tailored to disease subtypes.
